# SATB2-associated syndrome: characterization of skeletal features and of bone fragility in a prospective cohort of 19 patients

**DOI:** 10.1186/s13023-022-02229-5

**Published:** 2022-03-03

**Authors:** M. Mouillé, M. Rio, S. Breton, M. L. Piketty, A. Afenjar, J. Amiel, Y. Capri, A. Goldenberg, C. Francannet, C. Michot, C. Mignot, L. Perrin, C. Quelin, J. Van Gils, G. Barcia, V. Pingault, G. Maruani, E. Koumakis, V. Cormier-Daire

**Affiliations:** 1 Clinical Genetics, Necker Enfants Malades Hospital, APHP, 149 rue de Sevres, Paris, 75015 France; 2 Department of Neonatal Medicine, Cochin-Port Royal Hospital, APHP, Paris, France; 3 Department of Pediatric Radiology, Necker Enfants Malades Hospital, APHP, Paris, France; 4 Functional Exploration Laboratory, Necker Enfants Malades Hospital, APHP, Paris, France; 5 Sorbonne University, Reference Center for Intellectual Disabilities, Department of Genetics and Medical Embryology, Armand-Trousseau Hospital, APHP, Paris, France; 6 Clinical Genetics Functional Unit, Robert Debré Hospital, APHP, Paris, France; 7 Department of Clinical Genetics, Rouen, France; 8 Clinical Genetics, Clermont-Ferrand CHU, Clermont-Ferrand, France; 9 Paris Cité University, Reference Center for Constitutional Bone Diseases, INSERM UMR1163, Imagine Institute, Paris, France; 10 Clinical Genetics, La Pitié Salpétrière Hospital, APHP, Paris, France; 11 Clinical Genetics, Hospital Sud, Rennes, France; 12 Clinical Genetics, Hospital Pellegrin, Bordeaux, France; 13 Molecular Genetics, Necker Enfants Malades Hospital, APHP, Paris, France; 14 Reference Center for Skeletal Dysplasia, Cochin Hospital, APHP, Paris, France; 15 Department of Physiology, Hôpital Necker Enfants Malades and Hôpital Européen Georges Pompidou, AP-HP, Paris, France

**Keywords:** SATB2, Osteoporosis, ALP, Bone densitometry, X Ray, Demineralization

## Abstract

**Background:**

Individuals with pathogenic variants in *SATB2* display intellectual disability, speech and behavioral disorders, dental abnormalities and often features of Pierre Robin sequence. *SATB2* encodes a transcription factor thought to play a role in bone remodeling. The primary aim of our study was to systematically review the skeletal manifestations of SATB2-associated syndrome. For this purpose, we performed a non-interventional, multicenter cohort study, from 2017 to 2018. We included 19 patients, 9 females and 10 males ranging in age from 2 to 19 years-old. The following data were collected prospectively for each patient: clinical data, bone markers and calcium and phosphate metabolism parameters, skeletal X-rays and bone mineral density.

**Results:**

Digitiform impressions were present in 8/14 patients (57%). Vertebral compression fractures affected 6/17 patients (35%). Skeletal demineralization (16/17, 94%) and cortical thinning of vertebrae (15/17) were the most frequent radiological features at the spine. Long bones were generally demineralized (18/19). The distal phalanges were short, thick and abnormally shaped. C-telopeptide (CTX) and Alkaline phosphatase levels were in the upper normal values and osteocalcin and serum procollagen type 1 amino-terminal propeptide (P1NP) were both increased. Vitamin D insufficiency was frequent (66.7%).

**Conclusion:**

We conclude that *SATB2* pathogenic variants are responsible for skeletal demineralization and osteoporosis. We found increased levels of bone formation markers, supporting the key role of SATB2 in osteoblast differentiation. These results support the need for bone evaluation in children and adult patients with SATB2-associated syndrome (SAS).

**Supplementary Information:**

The online version contains supplementary material available at 10.1186/s13023-022-02229-5.

## Introduction

SATB2-associated syndrome (SAS, Glass syndrome, OMIM612313) is a rare autosomal dominant condition [1–4], related to *SATB2* mutations, characterized by intellectual disability, speech and behavioral disorders, dental abnormalities such as malposition and macrodontia and often features of Pierre Robin sequence with cleft palate*.*

*SATB2* (special AT rich sequence binding protein 2) is a highly conserved gene located on chromosome 2, (2q33.1,) 191 kb [1, 5]. It encodes a transcription factor involved in craniofacial, central nervous system and skeletal development. SATB2 is also thought to play a role in bone remodeling [6–8]. SATB2 is a regulator of Osx which in turn, is responsible for the differentiation of mesenchymal cells into osteoblasts. Indeed, SATB2 plays a role at 2 levels firstly by blocking Hoxa2, which controls negatively the differentiation of mesenchymal progenitor cells to pro-osteoblasts and secondly by stimulating the differentiation of osteoblasts [9]*.*

Several isolated clinical observations [10] have reported low bone mass, and/or bone fragility fracture in patients with *SATB2* variants [4]. More recently, Zarate el al described low bone mass and early-onset fragility fractures in a retrospective series of 7 patients with *SATB2* variants. In this series, elevated alkaline phosphatase levels were found in 5 individuals. To date, and to our knowledge, the characterization of skeletal involvement and of bone turnover markers in such patients has not yet been systematically collected in a prospective study. Moreover, the mechanism leading to early bone fragility is currently not completely understood. The aim of our study was to prospectively and systematically assess the skeletal manifestations observed in a cohort of 19 individuals with SAS, by the review of prevalent fractures and of skeletal X-rays. Patients underwent bone densitometry and had measurements of bone turnover markers as well as calcium and phosphate metabolism parameters.

## Subjects and methods

We performed a non-interventional, multicenter cohort study, from 2017 to 2018. Patients were recruited and reviewed via the French Reference Center for Constitutional Bone Diseases (CRMR MOC).

### Patients

19 patients with *SATB2* pathogenic variants were included in the study. Consent was signed. Patient data were anonymized.

#### Data collected

The following data were collected for each patient: (1) Clinical data including age, gender, height, weight, medical history, history of fracture(s), vitamin D supplementation and other ongoing or past antiosteoporotic treatments. (2) Biological tests including serum total and ionized calcium, phosphorus, albumin, ionized calcium, 25 hydroxy-vitamin D, 1,25 dihydroxy-vitamin D, serum parathyroid hormone (PTH). Alkaline phosphatase levels (ALP), osteocalcin and serum procollagen type 1 amino-terminal propeptide (P1NP) were chosen to assess bone formation, and C-telopeptide (CTX) was chosen as a marker of bone resorption. Because normal reference range of these tests differ with patient age, and to compare the results between patients, results were displayed as an ULM, i.e. as the ratio of (patient value)/ (upper limit of normal range). Importantly, evaluation was performed in the absence of  recent fracture (< 1 year). Radiological evaluation was perfomed in the context of standard care evaluation of their skeletal dysplasia for all patients. They were reviewed by an experienced radiologist specialized in skeletal dysplasias from the CRMR—MOC. X-rays from spine, skull, long bones, and hands/feet were read independently of the clinical, biological and bone densitometry phenotype. Areal bone mineral density (aBMD) was measured by dual-energy X-ray absorptiometry (DXA) at the lumbar spine (L1-L4), total hip, and femoral neck using LUNAR or HOLOGIC equipment and software. Z-scores were used for data analysis.

### Statistical analysis

Statistical analyses were performed using MedCalc Software (v11.6.1, MedCalc Software, Belgium) and Excel. Data are expressed as mean ± standard deviation (SD) for continuous variables, and numbers and percentages for categorical variables. Comparisons of subgroup characteristics were performed using Mann–Whitney test for unpaired data. Comparisons of proportions were performed using a chi-square test. Spearman’s rank correlation test was used to assess the relation between quantitative variables. For all analyses, a two-tailed *p*-value < 0.05 was considered to indicate statistical significance.

### Data protection

The study received approval from the committee for the protection of persons (CPP) on the 19/09/2017, file No 2017-102169-44, Reference CPP: 3534-NI. Data collection was in accordance with CNIL regulations and in accordance with the Helsinki declaration.

## Results

In all, 9 females (47%) and 10 males were included ranging in age from 2 to 19 years old. Extra skeletal features are summarized (Table 1). All mutations found were de novo, heterozygous and included partial or total deletion, missense and nonsense mutations (Table 2). None of our patients were taking medication.

**Table 1:** Extra skeletal features in the patients with *SATB2* variants

Patient	Features of Pierre Robin sequence	Dental abnormalities	Behavioral disorders	Intellectual disability	Speech disorders Speech delay
SATB2-01-P1	Yes	Hypodontia	No	Yes	Yes
SATB2-01-P2	No	Macrodontia	Autism spectrum disorder	Yes	Yes
SATB2-01-P3	No	Macrodontia	Autism spectrum disorder	Yes	Yes
SATB2-01-P4	No	Macrodontia	Autism spectrum disorder	Yes	Yes
SATB2-01-P5	No	Macrodontia	No	Yes	Yes
SATB2-01-P6	No	Macrodontia	No	Yes	Yes
SATB2-01-P7	Yes	Macrodontia	Autism spectrum disorder	Yes	Yes
SATB2-01-P9	No	Macrodontia	No	Yes	Yes
SATB2-01-P10	Yes	Macrodontia	Autism spectrum disorder	Yes	Yes
SATB2-01-P11	No	Macrodontia	No	Yes	Yes
SATB2-01-P12	Yes	Macrodontia	Autism spectrum disorder	Yes	Yes
SATB2-01-P13	Yes	Yes	No	Yes	Yes
SATB2-02-P1	No	Hypondotia	No	Yes	Yes
SATB2-04-P1	Yes	Yes	Yes	Yes	Yes
SATB2-05-P1	No	Yes	No	Yes	Yes
SATB2-06-P1	No	Yes	Autism spectrum disorder	Yes	Yes
SATB2-06-P2	No	Yes	Autism spectrum disorder	Yes	Yes
SATB2-07-P1	No	Yes	mild	Yes	Yes
SATB2-08-P1	No	No	No	Yes	Yes

**Table 2:** Clinical, molecular, biological and radiological details of the study

Patient	Gender	Age (y)	Mutation (NM_015265.3)	Clinical Fracture	Radiological fracture	Other X-ray findings	Lumbar Spine aBMD Z-score SD (g/cm2)	Total hip aBMD Z-score SD (g/cm2)	ALP ULM	CTX Plasm ULM	Ca 2.2–2.6 mmol/L	PTH 2^nd^ generation Roche 10–47 pg/ml	25 OH Vit D 30–60 ng/ml
SATB2-01-P1	Female	11	c.1197dup p.(Lys400*)	2 Humerus Clavicle		Thin Cranial vault with demineralization Digitiform impressions Spine demineralization with thick cortical bone Delayed maturation Small epiphyseal bones Metaphyseal striations Foliated cortical bone	− 3.5 (0.454)	− 2.7 (0.626)	NA	0.78	2.27	41	26
SATB2-01-P2	Male	10	c.1627del p.(Arg543Alafs*3)	1 Humerus	Dorsal Vertebral fracture	Cranial vault with demineralization Digitiform impressions Small epiphyseal bones Metaphyseal striations Foliated cortical bone	− 1,1 (0.492)	− 0.9 (0.627)	0.86	0.79	2.5	23.2	24
SATB2-01-P3	Male	7	Deletion exons 3 and 4	0	Vertebral fracture	Thin cranial vault with demineralization Digitiform impressions Thin spine cortical bone with demineralization Delayed maturation Metaphyseal striations Small epiphyseal bones Foliated cortical bone	− 1,8 (0.443)		1.09	0.81	2.31	50.4	21
SATB2-01-P4	Female	9	c.1165C > T (pArg389Cys)	1 Tibia		Spine with demineralization Delayed maturation Metaphyseal striations Small epiphyseal bones Foliated cortical bone	− 1.7 (0.447)	− 1.8 (0.488)	1.05	0.78	2.46	34.8	24
SATB2-01-P5	Male	11	Deletion exons 3 and 4	1 Humerus	Vertebral fracture	Cranial vault demineralization Digitiform impressions Thin spine and long bone cortical Metaphyseal striations Small epiphyseal bones Foliated cortical bone	− 2.8 (0.409)		0.78	1.05	2.5	64.4	26
SATB2-01-P6	Male	7	c.703G > T p.(Glu235*)	1 Clavicle	Vertebral fracture	Thin Cranial vault with demineralization Digitiform impressions Thin spine cortical bone with demineralization Delayed maturation Metaphyseal striations Small ivory epiphyseal bones Foliated cortical bone	− 0.9 (0.451)		0.80	0.81	2.51	42.2	24
SATB2-01-P7	Male	12	del 2q33.1 (minimum size of 29.114 bp) Deletion from 200,325,004 to 200,354,118 (GRCh37/hg19)	3 Vertebral fracture, thigh bone	Vertebral fracture	Thin spine cortical bone with demineralization Metaphyseal striations	− 2.0 (0,497)		0.68	NA	2.43	28.3	19
SATB2-01-P9	Male	4	c.715C > T p.(Arg239*)	0		Cranial vault demineralization Thin spine cortical bone with demineralization Delayed maturation Metaphyseal striations Small epiphyseal bones Foliated cortical bone	-2.6 (0,339)		0.78	0.89	2.45	20.8	42
SATB2-01-P10	Female	14	c.955C > T p.(Gln319*)	1 Forearm		Thin Cranial vault Spine with demineralization Delayed maturation Metaphyseal striations Foliated cortical bone	− 3.3 (0,746)		0.79	0.77	2.24	68.2	30
SATB2-01-P11	Male	4	c.715C > T p.(Arg239*)	0		Cranial vault demineralization			0.71	0.57	2.33	32.8	44
SATB2-01-P12	Male	1	c.658C > T p.(Gln220*)	0		Thin spine cortical bone with demineralization Skeletal maturation advance Foliated cortical bone			0.99	0.73	2.41	27.5	
SATB2-01-P13	Male	6	del2q31.3 (7.78 Mb)	0		Thin spine cortical bone with demineralization Delayed maturation Small epiphyseal bones Foliated cortical bone	− 2.6 (0,445)	− 0.9 (0,667)	NA	NA		31.2	25
SATB2-02-P1	Female	9	c.1198A > G p.(Lys400Glu)	0	Vertebral fracture	Cranial vault demineralization Digitiform impressions Thin spine cortical bone with demineralization Delayed maturation Metaphyseal striations Small epiphyseal bones Foliated cortical bone	0.5 (0,631)	− 1.1 (0,611)	1.27	NA	2.35	21	35
SATB2-04-P1	Female	7	c.715C > T p.(Arg239*)	0		Digitiform impressions Thin spine cortical bone with demineralization Delayed maturation Foliated cortical bone			1.12	NA	2.68	20.1	12.5
SATB2-05-P1	Female	12	c.1285C > T p.(Arg429*)	0		Cranial vault demineralization Digitiform impressions Spine with demineralization Metaphyseal striations	− 2.3 (0,619)	− 2.7 (0,572)	0.51	1.29		29.2	21
SATB2-06-P1	Female	13	c.1696G > A p.(Glu566Lys)	0		Thick Cranial vault with demineralization Thin spine cortical bone with demineralization Skeletal maturation advance Metaphyseal striations Small epiphyseal bones			0.23	0.19	2.47		22.74
SATB2-06-P2	Female	8	c.1010 dup p.(Phe339Alafs*13)	1 NA		Thin spine cortical bone with demineralization Small epiphyseal bones Foliated cortical bone			1.33	0.87	2.26	26	24
SATB2-07-P1	Female	10	c.1610del p.(Asn537Thrfs*9)	1 Elbow		Cranial vault with demineralization Thin spine cortical bone with demineralization Metaphyseal striations Small epiphyseal bones Foliated cortical bone			1.07	NA	2.42	19	37
SATB2-08-P1	Male	19	c.1538G > A p.(Ser513Asn)	0		Thin spine cortical bone	− 0.2 (0,992)	− 0.4 (1,094)	0.62	0.74	2.4	32	32.3

Bone densitometry results are expressed in g/cm2 and Z-score SD

*NA* not applicable, *SD* standard deviation

*ALP* alkaline phosphatase activity, *CTX* C-telopeptide, *ULM* upper limit method

### Skeletal features (Table 2)

Nine/nineteen (47%) patients had at least one reported clinical fracture. Of these 9 patients with fractures, one had a history of known vertebral fracture and 8 patients had peripheral fractures. Two patients had a history of more than one fracture, one of whom presented with a long bone deformity, vertebral and hip fractures and another patient with fractures of the humerus and clavicle. One patient had tibial bowing. Although the exact circumstances of fracture occurrence were not always known, they usually occurred following minor trauma and therefore were considered to be non-traumatic. None of the patients were receiving or had received bisphosphonates before the discovery of fractures.

### Skeletal evaluation

#### Calvaria (Table 3)

**Table 3:** Skeletal characteristics of the SATB2 cohort

**SATB2 cohort, 19 patients**	
Age (years ± SD)	9.2 (1–19 years)
Female gender (N, %)	9/19 (47%)
Clinically reported fractures (N, %)	9/19 (47%)
**Radiological fracture (N, %)**	
Fragility fracture (N, %)	6/17 (35%)
**Low bone mineral density at the lumber spine or total hip (N, %)**	
(Z-score ≤ − 2.0 SD)	7/17 (41%)
**Fragility fracture and/or Low bone mineral density**	
(Z-score ≤ − 2.0 SD) (N, %)	11/17 (65%)
**X-ray findings**	
*Calvaria*	
Digitiformes Impressions (N, %)	8/14 (57%)
Demineralization (N, %)	10/14 (71%)
*Spine*	
Vertebral compression fracture (N, %)	6/17 (35%)
Cortical fine (N, %)	15/17 (85%)
Demineralization (N, %)	16/17 (94%)
*Tubular bones*	
Demineralization (N, %)	18/19 (95%)
Streak metaphysis (N, %)	13/19 (68%)
Small epiphysis (N ,%)	12/19 (63%)
Cortical foliated (N, %)	15/19 (79%)
Cortical thick (N, %)	7/19 (37%)
*Hands*	
Distal phalanges shortened and widened (N, %)	7/16 (43%)
**aBMD**	
Lumbar spine Z-score (mean, SD)	− 1.81 ± 1.26
Total hip Z-score (mean, SD)	− 1.37 ± 0.94
**Biochemical findings**	
Total Serum Calcium (2.20–2.60, mean, ± SD)	2.41 ± 0.11
Serum Phosphate (mean, ± SD)	1.55 ± 0.35
Ionized Calcium *(1.1–1.3 mmol/L)*	1.24 ± 0.04
25 OH Vit D *(30–60 ng/ml)*	27.2 ± 8.1 SD
1,25 OH Vit D *(30–80 ng/l)*	64.96 ± 20.0 SD
ALP (UI/L)*	366.89 ± 114 SD (0.86 ± 0.28 ULM)
Osteocalcin (microg/l)*	92.40 (1.11 ULM)
P1NP (ng/ml)*	964.21 (1.65 ULM)
CTX (pmol/l)*	14221 (0.79 ULM)
PTH *(10–50 pg/ml)*	34 (0.74 ULM)

X Ray results as a percentage of a number of subjects. For DMO, results are given by the mean in g/cm^2^ and SD. For biological results, the mean of the measurements are given. For data whose reference ranges vary with age, we also calculated ULM (Upper Limit Method)

*For normal range, please refer to Additional files. Reference values are presented by age and gender. Section headings are in bold

Demineralization was observed in 10/14 (71%) patients. Thin calvarial bone was observed in 4/14 (29%) patients, whereas a thick calvaria was observed in 1/14 (7%) patients. Digitiform impressions were present in 8/14 (57%) patients.

#### Spine (Fig. 1 and Tables 2, 3)

**Fig. 1:**
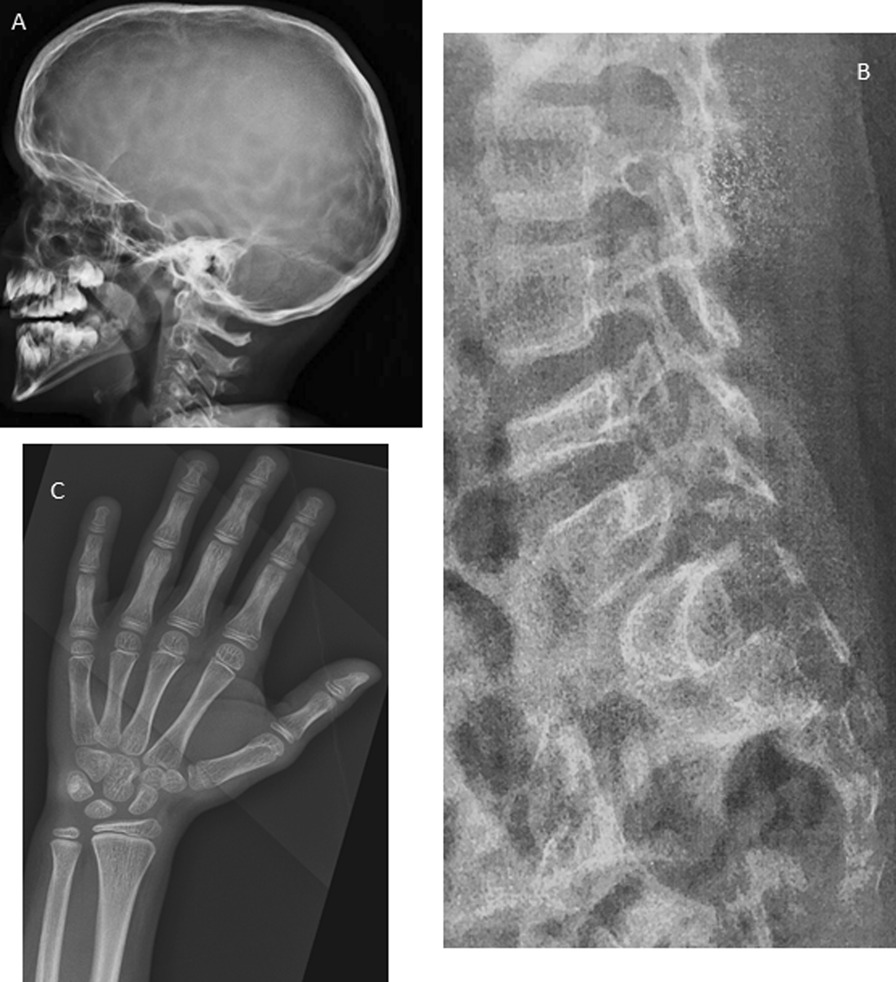
Radiological features. A. Skull X Ray (9 years 3 months girl). Note the thin cranial vault, the demineralization and the digitiform Impressions. B. Spine X Ray (10 years boy). Note the demineralization, the vertebral fracture, and the thin cortical bone. C. Hand X-Ray (9 years 3 months girl). Note the demineralization, the delayed bone age (age 6 years 10 months), small epiphyse, and striated metaphyses

Vertebral compression fractures were detected in 6/17 (35%) patients. Skeletal demineralization (16/17, 94%) and cortical thinning of vertebrae (15/17, 88%) were the most frequent features at the spine. Cortical collapse was seen in 2/17 (12%) patients*.* Long bones (Fig. 1 and Tables 2, 3)*.* Long bones were generally demineralized (18/19, 95%). The aspect of cortical bone was quite variable. No diaphyseal deformations or periosteal appositions were observed. A diaphyseal fracture of the femur was identified in one patient (5%). Cortical thickness was variable: normal (2/19, 11%), thick (7/19, 37%), and thin in 3/19 (16%) patients. Laminated bone cortex was frequently observed in 15/19 (79%).

#### Bone maturation (Fig. 1 and Tables 2, 3)

Delayed ossification of carpal bones was noted in half of the patients aged 3 to 13 years-old (9/18, 50%), but 2 patients (11%) aged 6 years to 13 years presented with advanced ossification of the carpal bones. Long bone striations were found at the metaphysis in 14/19 (68%) of cases. Small epiphyses were noted in 12/19 (63%) of cases and ivories in 2/19 (11%) of the cases.

#### Distal bones (Fig. 1)

The distal phalanges were short, thick and abnormally shaped (7/16, 43%).

#### Bone densitometry (Tables 2, 3)

Bone densitometry was available for 13/19 patients. Among these patients, 7/13 (54%) patients had a Z-score of − 2.0 SD or lower (Z-score ≤ − 2 SD) at the lumber spine or the total hip, and 5 / 13 (38%) patients had a Z-score between − 1 and − 2 SD. Mean lumbar spine Z-score was—1.9 ± 1.1SD. Mean hip Z-score was − 1.5 ± 0.9 SD. Bone mineral density was in the normal range for 3 patients (7, 9 and 19 years old).

#### Prevalence of osteoporosis (Table 3)

Although only one patient reported a history of vertebral compression fracture, a total of 6 patients (35%) had at least one vertebral compression fracture on X-ray findings; and 11 patients (65%) had a fragility fracture and/or aBMD Z-score ≤ -2 SD. The prevalence of osteoporosis defined by at least one vertebral compression fracture, or by the presence of both a clinically significant fracture history and a BMD Z-score ≤ -2.0 DS, was of 8/17 (47%).

### Biological evaluation (Tables 2, 3 and Additional file: Tables)

Serum total calcium, ionized calcium and serum phosphate were in the normal range in all patients. 25 OH Vitamin D value was below 30 ng/ml in 12/18 patients (66.7%), with a mean value of 27.2 ± SD 8.12 ng /mL. Parathyroid hormone (PTH) was above normal in 3 patients and in the normal range in most patients: 34.0 ± SD 14.42 pg/ml, min 19.0, max 68.2 (10–50 pg/ml), ie 0.74 ULM (Upper Limit Multiple) [11]. CTX levels were in the upper normal values when using reference range values adapted to the patient age: 14.221 ± SD 435 pmol / L, min 3364, max 18.832, (7.000–18.000 pmol / L), ie 0.79 ULM. Alkaline phosphatases (ALP) were above normal values in 10/17 patients (59%). Among patients with increased ALP values, 7/10 (70%) had a history of fractures but 30% had no history of clinical or radiological fracture. Osteocalcin and P1NP were both above normal values: 92.4 ± SD 34.7 microg / L, min 43.9, max 147 (38–83 microg / L), ie 1.11 ULM and 964.2 ± 381.8 ng / ml standard function of age, 1.65 ULM, respectively.

### Biochemical parameters and bone mineral density correlations (Table 4)

**Table 4:** Correlation between BMD and biological parameters

Hip BMD Z-score	Spine BMD Z-score	Phosphate	Ionized calcium	ALP	CTX	Osteocalcin	P1NP
**ALP**							
R = 0.334 *P* = 0.665	**R = 0.933** ***P*** **= 0.0204**		R = − 0.008 *P* = 0.9792		**R = 0.967** ***P*** **= 0.0331**	R = 0.779 *P* = 0.0676	**R = 0.687** ***P*** **= 0.0066**
**CTX**							
R = − 0.302 *P* = 0.4289	R = 0.254 *P* = 0.4793	R = 0.448 *P* = 0.1251	R = 0.387 *P* = 0.2141	**R = 0.967** ***P*** **= 0.0331**		**R = 0.678** ***P*** **= 0.0077**	**R = 0.549** ***P*** **= 0.0421**
**Osteocalcin**							
R = − 0.475 *P* = 0.1649	**R = **− **0.616** ***P*** **= 0.0331**	R = 0.044 *P* = 0.8759	R = 0.061 *P* = 0.8346	R = 0.779 *P* = 0.0676	**R = 0.678** ***P*** **= 0.0077**		R = 0.315 *P* = 0.2525
**P1NP**							
**R = **− **0.950** ***P*** **= 0.0132**	R = 0.585 *P* = 0.0588	**R = 0.847** ***P*** **= 0.0001**	R = − 0.141 *P* = 0.6466	**R = 0.687** ***P*** **= 0.0066**	**R = 0.549** ***P*** **= 0.0421**	R = 0.268 *P* = 0.3349	

Note that hip BMD is positively correlated with albuminemia and spine BMD, whereas it is negatively correlated with bone formation parameters osteocalcin and P1NP, and phosphate levels. Spine BMD is positively correlated with ionized calcium and ALP, and negatively correlated with osteocalcin. CTX is positively correlated with osteocalcin and ALP. Osteocalcin is positively correlated with CTX and negatively with spine BMD. P1NP is positively correlated with ALP. BMD is negatively correlated with markers of bone formation. Significant correlations are in bold

We next assessed whether bone mass was correlated with biochemical parameters. Hip BMD was positively correlated with spine BMD. Markers of bone formation (ALP and osteocalcin) were positively correlated with resorption markers (CTX) suggesting bone coupling between bone formation and bone resorption in patients with SAS. Bone formation marker ALP was positively correlated with P1NP and tended to be correlated with osteocalcin (*p* = 0.0676).

### Comparison of fractured with non-fractured SATB2 patients

ALP, assessed using the ULM levels, were significantly higher in individuals with pathogenic variants in SATB2 with a history of fracture compared to non-fractured patients (*p* = 0.01).

## Discussion

We report here the investigation of skeletal manifestations in a cohort of patients with *SATB2* variants. The SAS clinical spectrum described so far, has mainly focused on teeth abnormalities, developmental delay, speech limitations and behavioral issues. The aim of the study was to highlight the skeletal phenotype pertaining to premature osteoporosis. Indeed, we observed a particularly high frequency of fractures and low bone mass in these patients with 35% of radiologic vertebral compression fractures. Importantly, the frequency of vertebral compression fractures was very much underestimated with only one patient reporting vertebral fracture whereas 6/17 (35%) patients displayed radiographic vertebral fractures. Additionally, 54% of patients showed evidence of low bone mass as defined by a Z-score below − 2 SD.

Furthermore, the extensive radiological assessment of this cohort allows the description of unreported skeletal features including thin cortical bone, dysmorphic phalanges, and delayed carpal ossification.

The systematic biochemical assessment, performed independently of any fracture event, led to the observation of increased ALP levels in more than half the cohort (59%), using age adjusted reference range, as well as increased osteocalcin and P1NP levels, all known markers of bone formation. These findings support that the abnormalities in bone formation markers are the result of the underlying bone disease rather than related to a fracture event. We also observed a correlation between P1NP and ALP levels, in accordance with the increase in bone formation markers. These findings are consistent with previous report of increased ALP and/or P1NP and and/or osteocalcin in a very limited number of patients with SAS [1, 9]. Finally, we also found increased CTX levels, which were correlated with increased bone formation markers, supporting increased bone turnover in these patients [1].

The exact mechanism contributing to the skeletal phenotype remains incompletely understood. Bone formation markers are the products of active osteoblasts expressed during different phases of their development and considered to reflect different aspects of osteoblast function and bone formation. SATB2 regulates skeletogenesis by modulating the expression of osteoblast specific genes like *SOX9*, *ATF4* or *RUNX2*, and plays a role in the differentiation of osteoblasts [9]. Moreover, it has been also demonstrated that mutations in *SATB2* reduce pre-osteoblast formation supporting that SATB2 also regulates progenitor proliferation and participates in both proliferation and differentiation processes [12].

Although this study has limitations, including the small size of the cohort, and the difficulties in the interpretation of the bone markers values in children, related to age variability, it clearly highlights the broad spectrum of skeletal abnormalities, fractures and low bone mass in individuals with SAS. This study also supports the need for regular assessment of BMD and bone turnover markers in the management of patients with *SATB2* pathogenic variants. While none of the individuals reported here had any specific treatment apart from vitamin D supplementation, the role of SATB2 in osteoblast differentiation and proliferation may also suggest the use of anabolic agent targeting osteoblast. For this reason, we did not introduce bisphosphonates in this cohort. Furthermore, detecting any additional risk factor for osteoporosis and/or fractures, including vitamin D insufficiency, may help to prevent fractures in this context.

## Supplementary Information

**Additional file 1**. References for normal biological values for children and adults.

**Additional file 2**. Raw data and ULM values for P1NP and Osteocalcin in the 19 patients.

## Data Availability

The data are available in a local database and can be shared on request.
