# Supplementary material for: SATB2-associated syndrome: characterization of skeletal features and of bone fragility in a prospective cohort of 19 patients
Source: Orphanet J Rare Dis. 2022 Mar 3;17:100. doi: 10.1186/s13023-022-02229-5 (PMC8895909; doi:10.1186/s13023-022-02229-5)
Supplement: Supplementary file 1 — Additional file 1. References for normal biological values for children and adults. [file 13023_2022_2229_MOESM1_ESM.docx]

| **Female (*ng/ml)*** | | **Male *(ng/ml)*** | |
| --- | --- | --- | --- |
| **15 days -<1 year** | 122-469 | **15 days -<1 year** | 122-469 |
| **1 -10 years** | 142-335 | **1 -10 years** | 142-335 |
| **10-13 years** | 129-417 | **10-13 years** | 129-417 |
| **13-<15 years** | 57-254 | **13-<15 years** | 116-468 |
| **15-<17 years** | 50-117 | **15-<17 years** | 82-331 |
| **17-<19 years** | 45-87 | **17-<19 years** | 55-149 |
| **Adults** | 35-104 | **Adults** | 40-129 |

**Alkaline phosphatase** *(Cobas)***UI/L*:*** *Normal values for children and adults*

**Osteocalcin** *(Cobas)****ng/ml*:** *Normal values for children*

***Wyness et al. Clin Chim Acta 2013;415:169***

***Huang Y. et al. Clin Biochem 2011 ;44:771***

| **Female (*ng/ml)*** | | **Male *(ng/ml)*** | |
| --- | --- | --- | --- |
|  | 2.5-97.5 percentile |  | 2.5-97.5 percentile |
| **6 months – 1 year** | 44.9 – 122.5 | **6 months – 1 year** | 45.1 – 116.5 |
| **1 year** | 38.9 – 121.5 | **1 year** | 45.6 – 124.5 |
| **2 years** | 44.9 - 116 | **2 years** | 37.8 – 120.3 |
| **3 years** | 40.6 – 114.6 | **3 years** | 38.1 – 105.7 |
| **4 years** | 44.0 – 122.2 | **4 years** | 39.0 – 112.9 |
| **5 years** | 45.4 – 118.6 | **5 years** | 44.3 – 116.9 |
| **6 years** | 45.8 – 128.9 | **6 years** | 42.3 – 112.0 |
| **6 – 10 years** | 61.4 – 136.2 | **6 – 10 years** | 56.5 – 152.1 |
| **10 – 14 years** | 24.1 – 232.1 | **10 – 14 years**  **14 – 15 years** | 48.2 – 226.4 |
| **14 – 15 years** | 17.8 – 119.6 |  |  |
| **> 15 years** | 21.1 – 76.7 | **> 15 years** | 22.5 – 151.3 |

**Osteocalcin** *(Cobas)***:** Normal values for adults

| **Female *ng/ml*** | | **Male *ng/ml*** | |
| --- | --- | --- | --- |
| Age (a) | 10-90 percentile | Age | 10-90 percentile |
| **20 – 30** | 26.6 - 51 | **20 – 30** | 27.9 - 60 |
|  |  | **30 - 55** | 21.6 – 38.3 |
|  | 5-95 percentile |  | 5-95 percentile |
| **> 30** | 14.4 – 31.7 | **>55** | 15.0 – 35.61 |

**Cross-laps (CTX)** *(cobas)****pmol/L*:** *Normal values for children*

Wyness et al. Clin Chim Acta 2013; 415:169

De Melo et al. J Pediatr Endocrinol Metab 2018;31:637

| **Female** | | **Male** | |
| --- | --- | --- | --- |
| **<1 year** | 3131 - 11849 | **<1 year** | 2743 - 9292 |
| **6 months – 1 year** | 3177 - 13872 | **6 months – 1 year** | 3487 - 12632 |
| **1 year** | 3410 - 12400 | **1 year** | 4572 - 13252 |
| **2 years** | 4185 - 13640 | **2 years** | 4107 - 13175 |
| **3 years** | 4262 - 13795 | **3 years** | 4030 - 13252 |
| **4 years** | 4882 - 13950 | **4 years** | 3952 - 13175 |
| **5 years** | 3797 - 14570 | **5 years** | 5115 - 13795 |
| **6 years** | 3875 - 14260 | **6 years** | 4185 - 13020 |
| **6 – 9 years** | 4379 – 12167 | **6 – 9 years** | 3945 – 13152 |
| **10 – 13 years** | 4859 – 15151 | **10 – 13 years** | 6665 – 20080 |
| **14 – 18 years** | 2418 - 8556 | **12 – 14 years** | 8600 - 21500 |
|  |  | **15 – 17 years** | 5800 – 14000 |
|  |  | **18 – 19 years** | 4400 – 10700 |

**Cross-laps (CTX)** *(cobas)****pmol/L*:** *Normal values for adults*

*Jenkins N. et al. Bone 2013 ; 55 : 271*

| Age (years) | **femmes** | Age (years) | **hommes** |
| --- | --- | --- | --- |
|  | *2.5 – 97.5 percentile* |  | *2.5 – 97.5 percentile* |
| **< 30** | 1162 - 6200 | **25 - 40** | 1317 - 4650 |
| **30 - 39** | 775 - 5425 |  |  |
| **40 - 49** | 775 - 4650 | **40 - 60** | 1001 - 4650 |
| **> 50** | 775 - 5425 | **> 60** | 775 - 4650 |

**P1NP** *(Cobas)***, *ng/ml*:** *Normal values for children*

|  | | | **Female and male** | | | | |
| --- | --- | --- | --- | --- | --- | --- | --- |
| Age | | | Median | | Range (IC 95%) | | |
| **1 month- 1 year** | | | 631 | | 272 - 1919 | | |
| **1 year – 10 years** | | | 322 | | 190 - 548 | | |
| **Female** | | | | **Male** | | | |
| Age (years) | Median | range (IC 95%) | | Age (years) | | Median | range (IC 95%) |
| **10-13** | 362 | 187 - 790 | | **10-12** | | 269 | 165 - 423 |
| **13-14** | 297 | 103 - 651 | | **12-15** | | 499 | 193 - 1060 |
| **14-15** | 144 | 73 - 311 | | **15-16** | | 345 | 155 – 731 |
| **15-17** | 82 | 36 - 216 | | **16-19** | | 140 | 61 - 414 |
| **17-19** | 58 | 35 - 96 | |  | |  |  |

**P1NP** *(Cobas)***, *ng/ml*:** *Normal values for adults*

*Jenkins N et al. Bone 2013 ; 55 : 271*

| **Female** | | **Male** | |
| --- | --- | --- | --- |
| **< 30 years** | 25 - 90 | **25 – 70 years** | 15 - 80 |
| **30 – 39 years** | 15 - 80 |  |  |
| **40 – 49 years** | 15 - 60 |  |  |
| **50 – 69 years** | 15 - 75 |  |  |

**Wyness et al. Clin Chim Acta 2013 ; 415 : 169 (N = 1002) from 6 months to 6 years**

| P1NP, ng/ml | Male | | Female | |
| --- | --- | --- | --- | --- |
|  | 2.5 percentile | 97.5 percentile | 2.5 percentile | 97.5 percentile |
| 6 months- 1 year | 833.4 | 1856.2 | 903.4 | 1762.8 |
| 1 year | 643.3 | 1551.9 | 587.1 | 1504.7 |
| 2 years | 384.9 | 1139.6 | 421.1 | 1186.6 |
| 3 years | 338.9 | 870.1 | 327.7 | 969.8 |
| 4 years | 311.4 | 863.3 | 340.0 | 990.4 |
| 5 years | 329.7 | 863.5 | 344.7 | 787.9 |
| 6 years | 320.9 | 814.2 | 324.0 | 895.2 |

**Huang Y. et al. Clin Biochem 2011 ;44 :771 (N = 222, from 6 years to 18 years)**

| P1NP, ng/ml | Male | | Female | |
| --- | --- | --- | --- | --- |
|  | 2.5 percentile | 97.5 percentile | 2.5 percentile | 97.5 percentile |
| 6-10 years |  |  | 411 | 1022 |
| 10 - 11 years |  |  |  | 1451 |
| 11- 14 years |  |  | 109 | 1346 |
| 14 - 15 years |  |  | 38 | 510 |
| >15 years |  |  | 49 | 277 |
| 6 – 11 years | 407 | 1079 |  |  |
| 11- 14 years | 339 | 1399 |  |  |
| 14 – 15 years |  | 1217 |  |  |
| >15 years | 61 | 718 |  |  |
