# Supplementary material for: SATB2-associated syndrome: characterization of skeletal features and of bone fragility in a prospective cohort of 19 patients
Source: Orphanet J Rare Dis. 2022 Mar 3;17:100. doi: 10.1186/s13023-022-02229-5 (PMC8895909; doi:10.1186/s13023-022-02229-5)
Supplement: Supplementary file 2 — Additional file 2. Raw data and ULM values for P1NP and Osteocalcin in the 19 patients. [file 13023_2022_2229_MOESM2_ESM.docx]

| Patient | Gender | Age  (y) | P1NP  Raw data | P1NP  ULM | OC  Raw data | OC  ULM |
| --- | --- | --- | --- | --- | --- | --- |
| SATB2-01-P1 | Female | 11 | 1292.8 | 1.64 | 88.8 | 1.07 |
| SATB2-01-P2 | Male | 10 | 811.9 | 1.48 | 68.6 | 0.83 |
| SATB2-01-P3 | Male | 7 | 1064 | 1.94 | 79.2 | 0.95 |
| SATB2-01-P4 | Female | 9 | 1066 | 1.95 | 119.5 | 1.4 |
| SATB2-01-P5 | Male | 11 | 601.4 | 1.42 | 14.5 | 1.75 |
| SATB2-01-P6 | Male | 7 | 1047 | 1.91 | 78.8 | 0.95 |
| SATB2-01-P7 | Male | 12 |  |  |  |  |
| SATB2-01-P9 | Male | 4 | 1093 | 1.99 | 82.6 | 1 |
| SATB2-01-P10 | Female | 14 | 1142 | 1.75 | 131.3 | 1.6 |
| SATB2-01-P11 | Male | 4 | 816.9 | 1.49 | 51.9 | 0.63 |
| SATB2-01-P12 | Male | 1 | 1417.4 | *2.59* | 43.9 | 0.53 |
| SATB2-01-P13 | Male | 6 |  |  | 109.5 | 1.3 |
| SATB2-02-P1 | Female | 9 |  | 2.17 |  |  |
| SATB2-04-P1 | Female | 7 | 1189 | 1.72 | 102 | 1.2 |
| SATB2-05-P1 | Female | 12 | 1358 | 0.18 | 141.8 | 1.7 |
| SATB2-06-P1 | Female | 13 | 140.7 |  | 49.03 | 0.59 |
| SATB2-06-P2 | Female | 8 | 1193 | 2.18 | 147 | 1.8 |
| SATB2-07-P1 | Female | 10 | / |  | 63.4 | 0.76 |
| SATB2-08-P1 | Male | 19 | 230.1 | 0.29 | 68 | 0.82 |

ULM: upper limit method; OC: Osteocalcin

PINP and OC raw data ng/ml

**Supplementary table 2**

**Raw data and ULM values for P1NP and Osteocalcin in the 19 SAS patients**
